# Social support and frailty progression in community-dwelling older adults

**DOI:** 10.3389/fpubh.2024.1408641

**Published:** 2024-07-17

**Authors:** Muhammad Helmi Barghouth, Jessica Klein, Tim Bothe, Natalie Ebert, Elke Schaeffner, Nina Mielke

**Affiliations:** ^1^Institute of Public Health, Charité – Universitätsmedizin Berlin, corporate member of Freie Universität Berlin and Humboldt Universität zu Berlin, Berlin, Germany; ^2^Department of Social Policy and Social Security Studies, Hochschule Bonn-Rhein-Sieg, Sankt Augustin, Germany

**Keywords:** social determinants of health, epidemiology, biopsychosocial model of health, healthy aging, social support, frailty, older adults, frailty progression

## Abstract

**Objectives:**

Despite the growing evidence regarding the influence of social factors on frailty in older adults, the effect of social support remains unclear. This study aims to assess the association between social support and frailty progression (transition and incidence) in a sample of community-dwelling older adults.

**Methods:**

Using a cohort study design, 1,059 older adults from the Berlin Initiative Study were followed up for 2.1 years. Multinomial and logistic regression analyses were performed to assess the association of social support using Oslo Social Support Scale-3 with frailty transition and incidence, respectively. Gender differences were explored using stratified analyses.

**Results:**

At baseline, frailty prevalence in the study population [mean (SD) age 84.3 (5.6) years; 55.8% women] reached 33.1% with 47.0, 29.4 and 23.6% of the participants reporting moderate, strong and poor social support, respectively. Over the follow-up period, social support was not significantly associated with the frailty transition categories in the adjusted model. Conversely, the adjusted logistic regression analysis showed that participants with poor social support had twice the odds of becoming frail compared to those with strong social support (OR 2.07; 95% CI 1.08–3.95). Gender-stratified analyses showed comparable estimates to the main analysis but were statistically non-significant.

**Discussion:**

Our study results underpin the role of social factors in frailty incidence and highlight social support as a potential target for frailty-preventing interventions in older adults. Therefore, it is important to adopt a biopsychosocial model rather than a purely biomedical model to understand and holistically improve the health of community-dwelling older adults.

## 1 Introduction

Due to aging populations, frailty has become a considerable public health challenge (1). Frailty is characterized by loss of biological reserves and resistance to stressors resulting from cumulative declines across multiple physiologic systems with subsequent vulnerability to a range of adverse outcomes such as falls, disability, delirium, depression, hospitalization and premature mortality (2–5) with increased healthcare costs (6). It is commonly assessed using frailty phenotype by Fried whose prevalence in older adults aged 75 years and above reaches 18–46% (4). According to the frailty phenotype, individuals could be robust, prefrail or frail depending on the number of frailty criteria they fulfill (2). Frailty is commonly preceded by prefrailty, a prodromal phase that represents potentially reversible mild depletion of physiological reserves (7, 8). Prefrailty is more prevalent than frailty with prefrail individuals having a higher risk of transitioning to frailty than robust individuals (2, 9, 10). Frailty is a dynamic condition that could remain stable, worsen or even improve over time (11). This highlights the merit of exploring modifiable factors associated with its progression and their underlying mechanisms to develop interventions aiming to prevent frailty worsening or incidence (1).

Fostering healthy aging in community-dwelling older adults entails prevention of frailty through the preservation and enhancement of the individuals’ intrinsic physical and mental capacities as well as their interaction with their environments (12). Hence, the perspective on potential risk factors for frailty has developed into a comprehensive approach that includes socio-demographic factors as well as several biopsychosocial factors (13, 14). However, despite the increased adoption of a patient-centered approach to health management, research addressing social vulnerability and frailty in old age is still lacking (15). A pivotal determinant of the external environment and a universally acknowledged social determinant of health is social support (14, 16). It is defined as the perception and actuality that a person is cared for by others and is an esteemed and valued part of a social network (17). Social support prevents functional loss and impacts the physical and mental health of older adults through allowing them to cope with daily stressors, hence promoting their subjective well-being and healthy aging (18).

Social support is understood as part of social isolation which is also an emerging public health challenge since socially-isolated older adults are at an increased risk of several physical and psychological conditions (19, 20). A recent scoping review has shown that there is an association between social isolation and frailty in community-dwelling older adults (19); while another systematic review found inconclusive results (21). However, most studies adopted a cross-sectional design or did not provide information about the validity and reliability of utilized social support scales (19). Longitudinally, there is limited evidence on the association of social isolation and loneliness with frailty progression in older adults, thus warranting further research (19, 22). Moreover, in light of the culturally dependent perception of social support, it is important to highlight that studies conducted on this topic in European populations are limited in quantity, especially studies assessing social support using previously validated instruments (23). It is also debatable whether perceived social support differs between genders (24). Previous research addressing gender differences with regard to the association between social support and frailty reported mixed results (25, 26).

Given the significance of social support especially for community-dwelling older adults, the current analysis aims to investigate its contribution to frailty progression (transition and incidence) in this group; (1) the first research question addresses frailty transition in older adults – i.e., whether they improve, remain stable, worsen in frailty status or die – over the observation period, hypothesizing that the frailty status of those with poor social support at study baseline is more likely to worsen and less likely to improve over the observation period in comparison to others with strong or moderate social support; (2) the second research question addresses frailty incidence in non-frail older adults over the observation period, hypothesizing that those with poor social support at baseline are more likely to become frail over the follow-up period in comparison to others with strong or moderate social support.

Addressing these research questions aims to establish whether social support could be a viable target for interventions aiming to improve frailty status in frail older adults or prevent its incidence in those who are non-frail.

## 2 Methods

### 2.1 Study population

The current analysis utilized data from the Berlin Initiative Study (BIS). The BIS is a population-based cohort study of 2,069 community-dwelling older adults initially aiming to assess the incidence and progression of chronic kidney disease (CKD) in older adults over time with the goal of improving medical care provision for them with special focus on kidney health. Data collection commenced in 2009 and extended over 10 years by way of five biennial study visits (27). To be included in the study, participants had to be at least 70 years old and a member of the statutory health insurance fund *“AOK Nordost – Die Gesundheitskasse” (AOK)*. To assess CKD incidence and progression in older adults, individuals requiring nursing care or any kind of kidney replacement therapy such as dialysis as well as those who underwent kidney transplantation were not included in the BIS. All participants provided informed consent and the study was approved by the Charité – Universitätsmedizin Berlin ethics committee (EA2/009/08).

**Figure 1:**
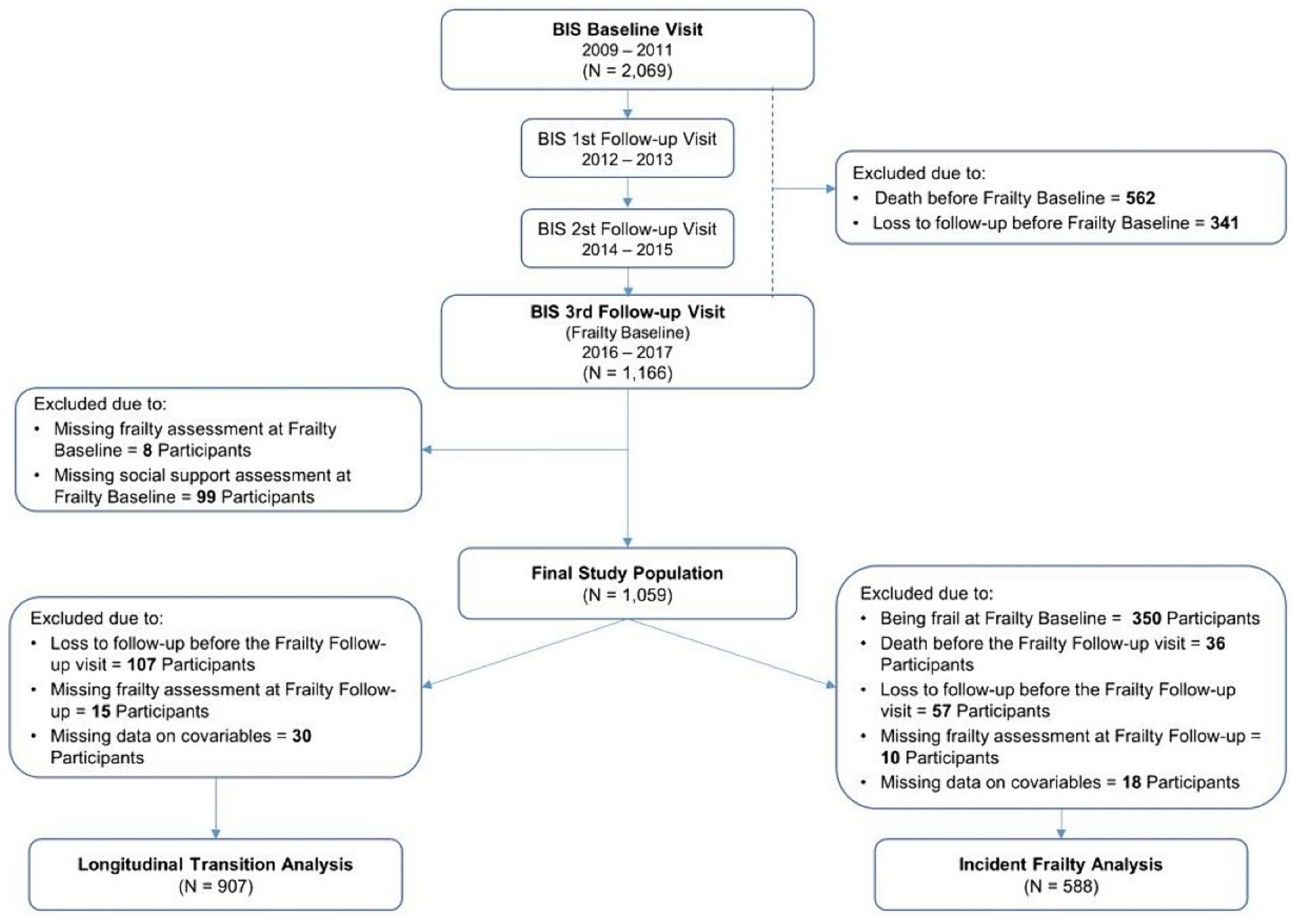
Overview of the Berlin Initiative Study (BIS) population. The flow chart shows the selection process and participants included in each of the research questions.

Frailty assessment was included in the study procedures of the third (between 2016 and 2017; hereinafter referred to as frailty baseline visit) and the fourth (between 2018 and 2019; hereinafter referred to as frailty follow-up visit) BIS follow-up visits. Hence, the current analysis included data from only those two study visits. Of the 1,166 individuals who participated at the frailty baseline visit, 1,059 participants with a valid assessment of their frailty status as well as their perceived social support at baseline were included (Figure 1).

### 2.2 Exposure: social support

Social support was measured using the Oslo Social Support Scale (OSSS-3) (24). This measure captures three aspects of perceived social support; (i) the number of people one can depend on during personal problems with the following response options (*none, 1–2, 3–5,* and *5+*); (ii) how much interest and concern the others show in one’s life with the following response options (*none, little, uncertain, some,* and *a lot*); and (iii) how easy it is to get practical help from neighbors when needed with the following response options (*very difficult, difficult, possible, easy,* and *very easy*). Scores of the individual questions are summed to create the total score ranging from 3 to 14 with higher scores indicating stronger social support. Based on the total score, social support is categorized into strong (12–14 points), moderate (8–11 points), and poor (3–7 points). The validity of OSSS-3 has been established with a Cronbach’s alpha value of 0.64 which is acceptable in light of the instrument’s brevity (24).

### 2.3 Outcome: frailty

Frailty was assessed using the modified Fried criteria (28); exhaustion, shrinking, and weakness which were adapted from Fried without modifications, slowness (requiring 15 s or more to complete the Timed Up and Go test), and finally, low physical activity (taking up physical activity exceeding 30 min less than once weekly). Participants were considered frail when they met at least three of the aforementioned criteria; prefrail when they met one or two criteria; and robust when they met none of the aforementioned criteria.

To address the first research question, five frailty transition categories were created. Participants were assigned to (1) the *stable non-frail category* when they were robust or prefrail and remained in the same category during both study visits; (2) the *stable frail category* when they were frail during both study visits; (3) the *improvement category* when they were frail at the frailty baseline visit and became either robust or prefrail at the frailty follow-up visit or prefrail at the frailty baseline visit and became robust at the frailty follow-up visit; (4) the *worsening category* when they were robust at the frailty baseline visit and became prefrail or frail at the frailty follow-up visit or prefrail at the frailty baseline visit and became frail during the frailty follow-up visit; and finally; and (5) the *death category* when they died before the frailty follow-up visit (28, 29).

Whereas for the second research question on frailty incidence, only non-frail participants (robust or prefrail) at frailty baseline were included. Incident frailty was operationalized as a dichotomous variable where participants who became frail during the observation period were categorized as incident frail and those who were robust or prefrail at the frailty follow-up visit remained in the non-frail category as done in previous studies (30–32).

### 2.4 Covariable assessment

Using a standardized computer-based questionnaire, primary data were collected on demographics, lifestyle variables, and comorbidities complemented by anthropometric and geriatric assessments. Moreover, primary data were augmented by individual level AOK claims data in which comorbidities were coded according to the 10th Revision of the International Classification of Diseases, German Modification (ICD-10) allowing the corroboration of self-reported data.

The following covariables were derived from the BIS data at the frailty baseline visit: age, gender, partner status as a dichotomous variable, self-rated health (SRH) as a three-category variable (*good, moderate,* and *poor*), general and vocational education as a three-category variable (*low, intermediate,* and *high*) according to the Comparative Analysis of Social Mobility in Industrial Nations (CASMIN) scale (33), body mass index (BMI) as a three-category variable (*≤22, 22–≤30,* and *>30 kg/m^2^*) as recommended by the Global Leadership Initiative on Malnutrition (34), and polypharmacy as a dichotomous variable which is defined as the regular intake of five or more prescription medications. Furthermore, multimorbidity was assessed using the Charlson Comorbidity Index (CCI) (35) based on the information derived from the AOK claims data.

### 2.5 Statistical analyses

Baseline characteristics of the study population stratified by social support categories were reported as absolute and relative frequencies for categorical variables, whereas for continuous variables, means and standard deviations (SD) or medians and interquartile ranges (IQR) were reported according to variable distribution.

To address the first research question, participants who were lost to follow-up before the frailty follow-up visit, did not have a valid frailty assessment at the frailty follow-up visit, or those with missing data regarding one or more covariables were further excluded from the initial study population resulting in the inclusion of 907 participants (Figure 1). Multinomial regression analysis was then conducted to assess the association between social support and frailty transition categories with the stable non-frail category as reference, and to estimate crude and adjusted relative risk ratios (RRR) and their corresponding 95% confidence intervals (95% CI). As for the second research question addressing frailty incidence in only non-frail participants, the previous exclusion criteria were applied followed by the further exclusion of participants who were already frail at the frailty baseline visit as well as those who died before the frailty follow-up visit yielding an analysis sample of 588 participants (Figure 1). Logistic regression analysis was conducted to assess the association between social support and incident frailty.

To address the potentially introduced selection bias through exclusion, the baseline characteristics of included and excluded participants were compared for each of the research questions separately. Both analyses were adjusted for the following knowledge-based set of covariates determined using a directed acyclic graph (36); age, gender, partner status, SRH, BMI, CASMIN, polypharmacy, and CCI. Possible gender differences were addressed through exploratory stratified analyses.

All analyses were conducted using R (Version 4.3.1; R Foundation for Statistical Computing, Vienna, Austria). Results were reported according to the Reporting of Observational Studies in Epidemiology (STROBE) statement (Supplementary A).

## 3 Results

### 3.1 Baseline characteristics of the study population

Table 1 shows the baseline characteristics of the study participants. The sample mean age (SD) was 84.3 (5.6) years and 55.8% were women. At baseline, most study participants reported moderate social support (47.0%), whereas participants with strong and poor social support comprised 29.4 and 23.6% of the study population, respectively. Some baseline variables showed a gradient across social support categories. With regard to gender, the proportion of women increased with decreasing social support ranging from 52.7 to 61.6%. Similarly, participants with poor social support had less often a partner compared to those reporting strong social support (39.6 vs. 57.6%). The proportion of participants reporting poor SRH increased with decreasing social support ranging from 11.6 to 21.2%, whereas the proportion of those reporting good SRH ranged from 53.1 to 39.2% with decreasing social support. Participants with weaker social support had also worse medical status indicators. Those with poor social support more often had five or more comorbidities (66.8%) and polypharmacy (53.2%) compared to their counterparts with strong social support (57.2 and 40.2%, respectively). At baseline, 33.1% of the total study population were frail. Frailty prevalence increased with age (Supplementary Table S1) and was also higher in participants with poor social support than in those with strong social support (38.0 vs. 26.4%).

**Table 1:** Baseline characteristics of the study population stratified by social support categories.

Variable	Category	Strong social support *N* = 311 (29.4%)	Moderate social support *N* = 498 (47.0%)	Poor social support *N* = 250 (23.6%)	Total *N* = 1,059
**Sociodemographic factors**					
Age Mean (SD)		84.3 (5.5)	84.4 (5.5)	84.2 (5.8)	84.3 (5.6)
Gender *N* (%)	Female	164 (52.7%)	273 (54.8%)	154 (61.6%)	591 (55.8%)
CASMIN *N* (%)	High	64 (20.6%)	111 (22.3%)	43 (17.2%)	218 (20.6%)
	Intermediate	65 (20.9%)	97 (19.5%)	50 (20.0%)	212 (20.0%)
	Low	181 (58.2%)	287 (57.6%)	156 (62.4%)	624 (58.9%)
	Missing	1 (0.3%)	3 (0.6%)	1 (0.4%)	5 (0.5%)
Partner status *N* (%)	Partnered	179 (57.6%)	258 (51.8%)	99 (39.6%)	536 (50.6%)
	Missing	1 (0.3%)	0	0	1 (0.1%)
Self-rated Health (SRH) *N* (%)	Good	165 (53.1%)	221 (44.4%)	98 (39.2%)	484 (45.7%)
	Moderate	110 (35.4%)	196 (39.4%)	99 (39.6%)	405 (38.2%)
	Poor	36 (11.6%)	79 (15.9%)	53 (21.2%)	168 (15.9%)
	Missing	0	2 (0.4%)	0	2 (0.2%)
**Medical status**					
Body Mass Index (BMI) (kg/m^2^) *N* (%)	≤22	26 (8.4%)	53 (10.6%)	26 (10.4%)	105 (9.9%)
	22–≤30	217 (69.8%)	329 (66.1%)	166 (66.4%)	712 (67.2%)
	>30	66 (21.2%)	109 (21.9%)	57 (22.8%)	232 (21.9%)
	Missing	2 (0.6%)	7 (1.4%)	1 (0.4%)	10 (0.9%)
Charlson Comorbidity Index (CCI) *N* (%)	Median (IQR)	6 (4, 9)	7 (5, 9)	7 (5, 9)	7 (4, 9)
	0	7 (2.3%)	13 (2.6%)	7 (2.8%)	27 (2.5%)
	1–2	44 (14.1%)	64 (12.9%)	27 (10.8%)	135 (12.7%)
	3–4	78 (25.1%)	101 (20.3%)	48 (19.2%)	227 (21.4%)
	≥5	178 (57.2%)	311 (62.4%)	167 (66.8%)	656 (61.9%)
	Missing	4 (1.3%)	9 (1.8%)	1 (0.4%)	14 (1.3%)
Polypharmacy *N* (%)	Yes	125 (40.2%)	239 (48.0%)	133 (53.2%)	497 (46.9%)
	Missing	1 (0.3%)	6 (1.2%)	1 (0.4%)	8 (0.8%)
Frailty status *N* (%)	Robust	69 (22.2%)	101 (20.3%)	44 (17.6%)	214 (20.2%)
	Prefrail	160 (51.4%)	224 (45.0%)	111 (44.4%)	495 (46.7%)
	Frail	82 (26.4%)	173 (34.7%)	95 (38.0%)	350 (33.1%)

Percentages of the social support categories are row percentages, whereas those of the individual variables are column percentages.

CASMIN, Comparative Analysis of Social Mobility in Industrial Nations; SRH, Self-rated Health; BMI, Body Mass Index; CCI, Charlson Comorbidity Index; IQR, Interquartile Range.

### 3.2 Social support and frailty transition

**Figure 2:**
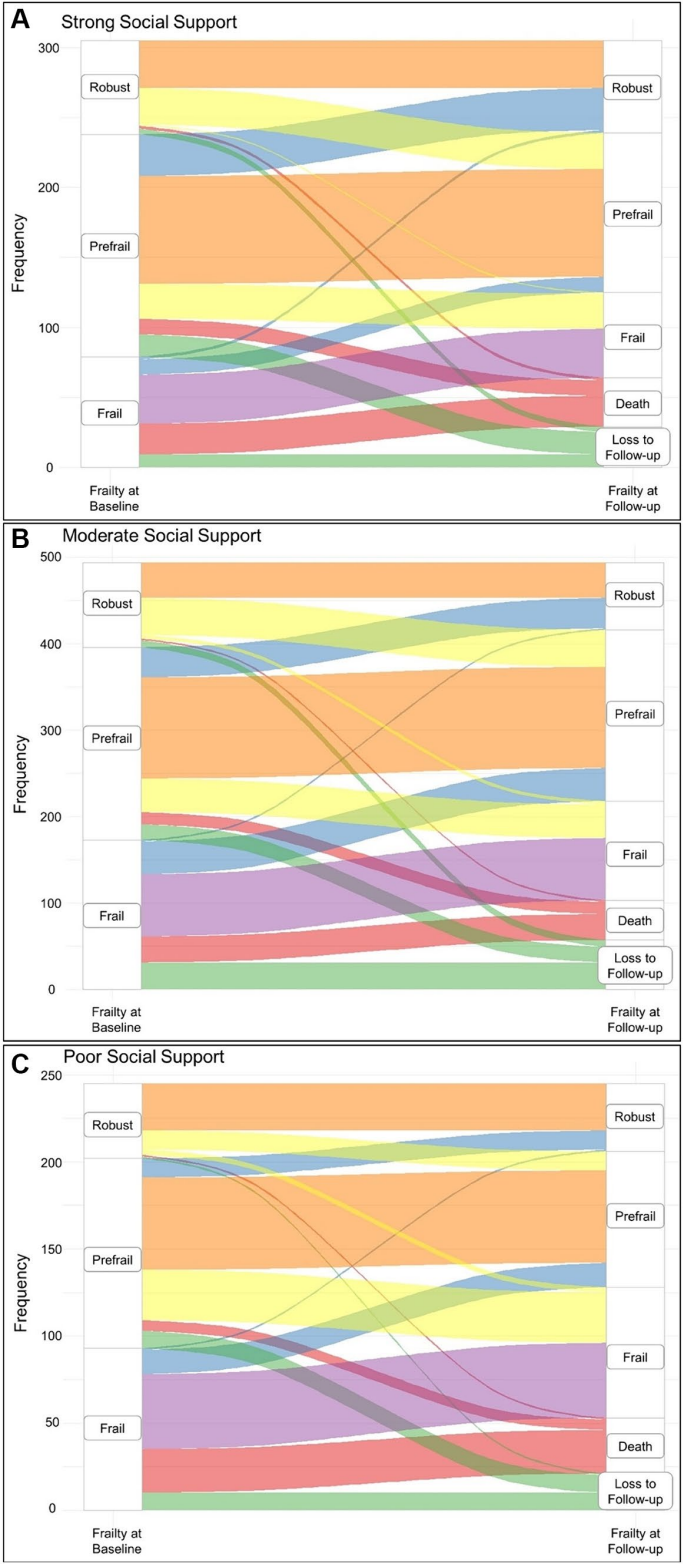
Sankey diagram showing the frailty transition categories over the observation period stratified by social support categories at baseline. **(A)** Frailty transitions in participants with strong social support. **(B)** Frailty transitions in participants with moderate social support. **(C)** Frailty transitions in participants with poor social support. The color coding refers to the frailty transition categories; orange corresponds to the *stable non-frail category*, yellow corresponds to the *worsening category*, blue corresponds to the *improvement category*, purple corresponds to the *stable frail category*, red corresponds to the *death category*, and finally, green corresponds to the *lost to follow-up group*.

Over a median follow-up period of 2.1 (IQR 2.0–2.3) years, the majority of study participants remained stable in frailty status across all three categories of social support (Table 2 and Figure 2). However, the proportion of participants in the stable frailty transition categories was highest among those with strong social support and lowest among those with poor social support (73.1 vs. 64.6% respectively) (Table 2). Frailty status improved more often in the moderate social support category (9.1%) than in the strong and poor social support categories (4.9 and 6.8% respectively). The proportions of participants whose frailty status worsened and those who died were highest in the poor social support category (14.1 and 14.5% respectively).

**Table 2:** Multivariable multinomial regression model showing the association between social support and frailty transition categories in the total population.

	Total *N* = 907	Frailty transition categories				
		Stable non-frail	Stable frail	Improvement	Worsening	Death
**Number of events *N* (%)**						
Social support						
Strong	268	162 (60.4%)	34 (12.7%)	13 (4.9%)	25 (9.3%)	34 (12.7%)
Moderate	419	228 (54.4%)	67 (16.0%)	38 (9.1%)	42 (10.0%)	44 (10.5%)
Poor	220	100 (45.5%)	42 (19.1%)	15 (6.8%)	31 (14.1%)	32 (14.5%)
**Crude model RRR (95% CI)**						
Social support						
Moderate		Reference	1.37 (0.85–2.22)	1.18 (0.75–1.85)	1.13 (0.74–1.73)	0.90 (0.54–1.50)
Poor		Reference	1.67 (0.98–2.86)	0.84 (0.48–1.48)	1.09 (0.66–1.80)	1.28 (0.73–2.24)
**Adjusted model RRR (95% CI)**						
Social support						
Moderate		Reference	1.06 (0.61–1.84)	1.11 (0.70–1.77)	1.05 (0.68–1.63)	0.82 (0.46–1.46)
Poor		Reference	1.24 (0.66–2.35)	0.76 (0.42–1.37)	0.98 (0.58–1.66)	1.28 (0.67–2.45)

The percentages in the descriptive part of the table are row percentages.

The model is adjusted for age, gender, partner status, body mass index (BMI), Comparative Analysis of Social Mobility in Industrial Nations (CASMIN), self-rated health (SRH), polypharmacy, Charlson Comorbidity Index (CCI).

Loss to follow up of participants over the observation period was very similar across the exposure categories ranging from 8 to 10% (Figure 2).

With regard to the first research question, multinomial regression showed that participants in the poor social support category had comparably elevated risks of being in the stable frail (adjusted RRR 1.24; 95% CI 0.66–2.35) and death (adjusted RRR 1.28; 95% CI 0.67–2.45) categories. Conversely, they had lower risks of being in the improvement category (adjusted RRR 0.76; 95% CI 0.42–1.37). A comparison of baseline characteristics between the included participants and the total study population was not significant. Moreover, the distribution of the exposure between included and excluded participants was very similar (Supplementary Table S2).

### 3.3 Social support and incident frailty

As for the second research question, participants at frailty baseline were restricted to those who were non-frail (709 participants). After 2.1 years, 101 (14.3%) participants became frail and 36 (5.1%) died.

The baseline characteristics across inclusion status were very similar (Supplementary Table S3). Despite the excluded participants being older with a higher frequency of multimorbidity, their distribution across social support categories was similar. Of the included non-frail participants with poor social support at baseline (*N* = 131), 31 (23.7%) became frail (Table 3), whereas 42 (15.6%) and 25 (13.4%) participants became frail among participants with moderate (*N* = 270) and strong (*N* = 187) social support at baseline, respectively. Having poor social support was associated with twice the odds of becoming frail (adjusted OR 2.07; 95% CI 1.08–3.95), while moderate social support was not associated with a significant increase in the odds of becoming frail (adjusted OR 1.16; 95% CI 0.66–2.06) over the observation period.

**Table 3:** Multivariable logistic regression model showing the association between social support and incident frailty in the total population.

	Total *N* = 588	Number of events *N* (%)	Crude model OR (95% CI)	Adjusted model OR (95% CI)
**Social support**				
Strong	187	25 (13.4%)	Reference	Reference
Moderate	270	42 (15.6%)	1.19 (0.70–2.04)	1.16 (0.66–2.06)
Poor	131	31 (23.7%)	2.01 (1.12–3.60)	2.07 (1.08–3.95)

The model is adjusted for age, gender, partner status, body mass index (BMI), Comparative Analysis of Social Mobility in Industrial Nations (CASMIN), self-rated health (SRH), polypharmacy, Charlson Comorbidity Index (CCI).

### 3.4 Gender differences

Gender-stratified analyses for both research questions showed comparable effect estimates to those of the respective total population; however, the corresponding CIs became much wider rendering them statistically non-significant (Supplementary Tables S4–S7). Nevertheless, the results of gender-stratified analyses should be interpreted with caution due to the relatively low number of events.

## 4 Discussion

In this study of social support and frailty in older adults, most study participants reported having moderate social support (47.0%), followed by strong social support (29.4%), and finally, poor social support (23.6%). The frailty prevalence in our study reached 33.1% at baseline. Over the observation period, frailty transitions did not differ significantly across social support categories. Conversely, non-frail participants with poor social support had twice the odds of becoming frail over the observation period in comparison to their counterparts with strong social support. These results did not differ significantly between genders.

The frequency distribution of social support in our study population was comparable to that of the general German population using the same validated instrument (strong 29.4 vs. 30.3%, moderate 47.0 vs. 45.3%, poor 23.6 vs. 24.2%) with a comparable mean (SD) OSSS-3 score for individuals aged 75 years and above [10.1 (2.2) vs. 9.8 (2.4)] (24).

Regarding frailty, its prevalence depends on the age of the population and the assessment tool used (4). Our prevalence was similar to that previously reported in a German sample with comparable mean age (37). The age-specific frailty prevalence in our study of 25% among individuals aged 80–84 years was also comparable to that of the same age group in Australia, Finland and China (38–40).

Frailty transition rates vary depending on study observation periods (22). A study conducted in China reported comparable transition rates to our study (improvement 16.6 vs. 15.3%, worsening 19.1 vs. 19.3%, stable 54.6 vs. 53.3%, and death 9.7 vs. 12.1%) over the same observation period of 2 years (41). When assessing the association between social support and frailty transition, our results show modest and non-significant effect estimates. Results from other studies conducted in the European context were mixed. Social isolation was not found to be associated with frailty transition over 6 years in an English study with a mean population age of 69.3 years (25). Conversely, it was found to be significantly associated with frailty trajectories in the same cohort over a 14-year period (42). It was also found to be associated with frailty worsening but not improvement in a European multinational cohort with a mean age of 70.5 years over a 2-year observation period (43). Studies conducted in Asian countries also reported mixed results showing no association between social factors and frailty transition trajectories (44) or an association between social activity and frailty improvement (45).

The varying results could be attributable to the use of different instruments to measure frailty (46) and its different operationalisations, the variation of constructs underlying social support in different studies, and subsequently, the use of various – sometimes non-validated – instruments for its measurement (19). Further influencing factors could be the difference in observation periods (22, 47), sample mean ages, and finally, the potential confounders adjusted for as comorbidities – which were reported to be associated with social support in older adults (48) – were not adjusted for in some studies (16, 42).

On the other hand, incident frailty was found to be significantly associated with poor social support in our study. These results are in agreement with those reported in an English study as well as by studies conducted in Singapore and Japan (16, 26, 42). This consistently significant association between social support and frailty incidence across contexts despite the previous considerations points to its robustness and relevance.

Frailty is a multifactorial condition with closely interlinked physiological and psychosocial components through which social support is believed to exert its impact and these pathways sometimes reciprocally influence social support as well (49, 50).

Physiologically, social support is assumed to prevent frailty worsening through reduction of disease burden (50). More specifically, a stress buffering effect of stronger social support was described through lowered cardiovascular reactivity (51). The resultant lower resting blood pressure subsequently hampers the decline of kidney function associated with frailty incidence and worsening in old age (29, 52). Moreover, frailty incidence is thought to be facilitated by chronic inflammation mediated by inflammatory cytokines whose levels were found to be lower in individuals with stronger social support (29, 53). The role played by these pathophysiological processes in developing frailty could partly explain the lack of association between social support and frailty transition in our study after adjusting for comorbidities. On the other hand, social support could impact frailty through a psychological and behavioral pathway as it could promote healthy behavior and better medication adherence (50). The negative impact of this pathway is possibly mediated by depression which was described as psychosocial frailty (49). Depression was found to coexist with and to aggravate physical frailty as well as to predict its incidence in individuals with cerebrovascular disease commonly found in older adults (49, 54, 55). Furthermore, depression was associated with a higher risk of fatigue and sarcopenia leading to less engagement in physical activity (56).

Social support and the level of social participation may be negatively affected in frail older adults due to multimorbidity and depression which highlights that the link between social support and frailty through the aforementioned pathways is not unidirectional, but exists more so in a feedback loop (50, 57).

The concept of social support and how it contributes to buffering the damaging effects of stress varies widely depending on one’s cultural background (23, 58). Hence, cross-cultural comparisons should be done with caution as generalizability of results is limited. This underscores the need to consider the evidence generated within culturally-comparable contexts and assessed using culturally-specific social support instruments to avoid construct bias (59). This is especially relevant with respect to gender differences in the association between social support and frailty. In our analysis, the effect estimates differed slightly, albeit non-significantly between genders. Perceived social support is believed to vary between individuals due to their biological sex-dependent socialization which is continuously changing to varying degrees in different cultures (24, 60). However, such variation could be mediated by other factors such as educational level or partner status (61, 62), which could partly explain the lack of gender differences in our results when these factors are adjusted for. This warrants adopting an intersectional approach regarding gender and culture and a nuanced depiction of gender differences in other social dimensions such as social ties and participation.

This study has several strengths. Social support was assessed using an instrument that was validated in the German population (24), and measured self-reported perceived social support which is sensitive to how far individuals are able to cope with stresses (63). This study adopted a longitudinal design clearly outlining the temporal relationship between social support and frailty. We operationalized frailty transition comprehensively by including death as a transition category. Our study population is representative for its source population of the *AOK Nordost* insurance fund which includes the largest proportion of older adults. Finally, through the combination of primary data with complementary claims data, we were able to adjust for several relevant confounding factors. The study results should also be interpreted against the backdrop of some limitations. The limited observation period may not have been sufficient to show the impact of social support and frailty transition as physical factors such as multimorbidity may have a more salient role on frailty transition on the short-term than social support. Also, the used frailty instrument considered only frailty phenotype – reflecting only physical frailty – and not varying severities of frailty, hence other dimensions of frailty as well as transition between frailty levels within frail participants could not be investigated. Further, this study could not consider the impact of other social isolation aspects such as social participation. Finally, we were not able to assess the mediating effect of depression as its assessment based solely on claims data leads to inaccurate estimates of its prevalence (64).

In conclusion, our results help identify social support as a viable target for interventions aiming to prevent frailty incidence and promote healthy aging in older adults. Strengthening social support of older individuals is advisable to promote their psychosocial well-being, foster health-promoting behavior and improve their physical condition. Empowering older adults by including them and considering their preferences is crucial when planning activities to strengthen their social support and increase participation. Furthermore, future research using validated social support instruments as well as multidimensional frailty instruments over a longer observation period is required to verify the robustness of our results.

## Data availability statement

The datasets presented in this article are not readily available because the data used in this study cannot be made available in the manuscript, the Supplementary files, or in a public repository due to German data protection laws (Bundesdatenschutzgesetz). To facilitate the replication of results, the used data will be stored on a secure drive at Charité – Universitätsmedizin Berlin. Access to the raw data used in this study can only be provided to external parties under the conditions of a cooperation contract and can be accessed upon request, after written approval. Requests to access the datasets should be directed to bis@charite.de.

## Ethics statement

The studies involving humans were approved by Ethics Committee of the Charité – Universitätsmedizin Berlin. The studies were conducted in accordance with the local legislation and institutional requirements. The participants provided their written informed consent to participate in this study.

## Author contributions

MB: Conceptualization, Formal analysis, Methodology, Visualization, Writing – original draft, Writing – review & editing. JK: Methodology, Writing – review & editing. TB: Writing – review & editing. NE: Data curation, Writing – review & editing. ES: Data curation, Funding acquisition, Project administration, Writing – review & editing. NM: Conceptualization, Data curation, Formal analysis, Methodology, Supervision, Writing – review & editing.

## Supplementary material

### Abbreviations

AOK, Statutory health insurance fund AOK Nordost – Die Gesundheitskasse; BIS, Berlin Initiative Study; BMI, Body mass index; CASMIN, Comparative Analysis of Social Mobility in Industrial Nations scale; CCI, Charlson Comorbidity Index; CKD, Chronic kidney disease; ICD-10, 10th Revision of the International Statistical Classification of Diseases and Related Health Problems; IQR, Interquartile range; OR, Odds ratio; OSSS-3, Oslo Social Support Scale-3; RRR, Relative risk ratio; SD, Standard deviation; SRH, Self-rated health; STROBE, Strengthening the Reporting of Observational Studies in Epidemiology.
