# Supplementary material for: Social support and frailty progression in community-dwelling older adults
Source: Front Public Health. 2024 Jul 17;12:1408641. doi: 10.3389/fpubh.2024.1408641 (PMC11288939; doi:10.3389/fpubh.2024.1408641)
Supplement: Supplementary file 1 [file Data_Sheet_1.docx]

**Supplement**

**Supplement A:** STROBE Statement

**Table S1:** The distribution of the frailty status of the study population stratified by age groups.

**Table S2:** Research question 1: Baseline characteristics stratified by inclusion status

**Table S3:** Research question 2: Baseline characteristics stratified by inclusion status

**Table S4:** Multivariable multinomial regression model showing the association between social support and frailty transition categories in men.

**Table S5:** Multivariable logistic regression model showing the association between social support and incident frailty in men.

**Table S6:** Multivariable multinomial regression model showing the association between social support and frailty transition categories in women.

**Table S7:** Multivariable logistic regression model showing the association between social support and incident frailty in women.

**Supplement A:**

Checklist of items that should be reported in cohort studies

|  | Item No | Recommendation | Section |  |
| --- | --- | --- | --- | --- |
| **Title and abstract** | 1 | (*a*) Indicate the study’s design with a commonly used term in the title or the abstract | Abstract |  |
|  |  | (*b*) Provide in the abstract an informative and balanced summary of what was done and what was found | Abstract |  |
| Introduction | | |  | |
| Background/rationale | 2 | Explain the scientific background and rationale for the investigation being reported | Introduction |  |
| Objectives | 3 | State specific objectives, including any prespecified hypotheses | Introduction – Last paragraph |  |
| Methods | | |  | |
| Study design | 4 | Present key elements of study design early in the paper | Methods – Study population |  |
| Setting | 5 | Describe the setting, locations, and relevant dates, including periods of recruitment, exposure, follow-up, and data collection | Methods – Study population |  |
| Participants | 6 | (*a*) Give the eligibility criteria, and the sources and methods of selection of participants. Describe methods of follow-up | Methods – Study population |  |
|  |  | (*b*) For matched studies, give matching criteria and number of exposed and unexposed | Not applicable |  |
| Variables | 7 | Clearly define all outcomes, exposures, predictors, potential confounders, and effect modifiers. Give diagnostic criteria, if applicable | Methods –  Exposure: Social support, Outcome: Frailty, Covariable assessment |  |
| Data sources/ measurement | 8* | For each variable of interest, give sources of data and details of methods of assessment (measurement). Describe comparability of assessment methods if there is more than one group | Methods –  Exposure: Social support, Outcome: Frailty, Covariable assessment |  |
| Bias | 9 | Describe any efforts to address potential sources of bias | Methods – Statistical analyses |  |
| Study size | 10 | Explain how the study size was arrived at | Methods – Study population, statistical analyses |  |
| Quantitative variables | 11 | Explain how quantitative variables were handled in the analyses. If applicable, describe which groupings were chosen and why | Methods – Statistical analyses |  |
| Statistical methods | 12 | (*a*) Describe all statistical methods, including those used to control for confounding | Methods – Statistical analyses |  |
|  |  | (*b*) Describe any methods used to examine subgroups and interactions |  |  |
|  |  | (*c*) Explain how missing data were addressed |  |  |
|  |  | (*d*) If applicable, explain how loss to follow-up was addressed |  |  |
|  |  | (*e*) Describe any sensitivity analyses |  |  |
| Results | | |  | |
| Participants | 13* | (a) Report numbers of individuals at each stage of study—eg numbers potentially eligible, examined for eligibility, confirmed eligible, included in the study, completing follow-up, and analysed | Figure 1 |  |
|  |  | (b) Give reasons for non-participation at each stage |  |  |
|  |  | (c) Consider use of a flow diagram |  |  |
| Descriptive data | 14* | (a) Give characteristics of study participants (eg demographic, clinical, social) and information on exposures and potential confounders | Results – Baseline characteristics of the study population, Table 1 |  |
|  |  | (b) Indicate number of participants with missing data for each variable of interest |  |  |
|  |  | (c) Summarise follow-up time (eg, average and total amount) |  |  |
| Outcome data | 15* | Report numbers of outcome events or summary measures over time | Results – Tables 2 & 3 |  |
| Main results | 16 | (*a*) Give unadjusted estimates and, if applicable, confounder-adjusted estimates and their precision (eg, 95% confidence interval). Make clear which confounders were adjusted for and why they were included | Results – Social support and frailty transition, Social support and incident frailty |  |
|  |  | (*b*) Report category boundaries when continuous variables were categorized |  |  |
|  |  | (*c*) If relevant, consider translating estimates of relative risk into absolute risk for a meaningful time period |  |  |
| Other analyses | 17 | Report other analyses done—eg analyses of subgroups and interactions, and sensitivity analyses | Results – Gender differences  Supplement tables 4-7 |  |
| Discussion | | |  | |
| Key results | 18 | Summarise key results with reference to study objectives | Discussion – First paragraph |  |
| Limitations | 19 | Discuss limitations of the study, taking into account sources of potential bias or imprecision. Discuss both direction and magnitude of any potential bias | Discussion – Penultimate paragraph |  |
| Interpretation | 20 | Give a cautious overall interpretation of results considering objectives, limitations, multiplicity of analyses, results from similar studies, and other relevant evidence | Discussion |  |
| Generalisability | 21 | Discuss the generalisability (external validity) of the study results | Discussion |  |
| Other information | | |  | |
| Funding | 22 | Give the source of funding and the role of the funders for the present study and, if applicable, for the original study on which the present article is based | Title page |  |

Table S1: The distribution of the frailty status of the study population stratified by age groups.

|  | | **Frailty Status** | | |
| --- | --- | --- | --- | --- |
|  |  | **Robust** | **Prefrail** | **Frail** |
| **Age Groups** | **75-79** | 107 (35.9%) | 142 (47.7%) | 49 (16.4%) |
|  | **80-84** | 75 (22.5%) | 175 (52.4%) | 84 (25.1%) |
|  | **85-89** | 22 (9.3%) | 114 (48.3%) | 100 (42.4%) |
|  | **≥90** | 10 (5.2%) | 64 (33.5%) | 117 (61.3%) |
|  | **Total** | 214 (20.2%) | 495 (46.7%) | 350 (33.1%) |

Table S2: Research question 1: Baseline characteristics stratified by inclusion status

| **Variable** | **Category** | **Included** | **Excluded** | **Total** N = 1,059 |
| --- | --- | --- | --- | --- |
|  |  | N = 907 (85.6%) | N = 152 (14.4%) |  |
| **Sociodemographic factors** | | | | |
| **Age**  Mean (SD) | | 84.3 (5.6) | 84.6 (5.6) | 84.3 (5.6) |
| **Gender**  N (%) | Female | 509 (56.1%) | 82 (53.9%) | 591 (55.8%) |
| **Social Support** N (%) | Strong | 268 (29.5%) | 43 (28.3%) | 311 (29.4%) |
|  | Moderate | 419 (46.2%) | 79 (52.0%) | 498 (47.0%) |
|  | Poor | 220 (24.3%) | 30 (19.7%) | 250 (23.6%) |
| **CASMIN** N (%) | High | 183 (20.2%) | 35 (23.0%) | 218 (20.6%) |
|  | Intermediate | 186 (20.5%) | 26 (17.1%) | 212 (20.0%) |
|  | Low | 538 (59.3%) | 86 (56.6%) | 624 (58.9%) |
|  | Missing | 0 | 5 (3.3%) | 5 (0.5%) |
| **Partner status** N (%) | Partnered | 464 (51.2%) | 72 (47.4%) | 536 (50.6%) |
|  | Missing | 0 | 1 (0.7%) | 1 (0.1%) |
| **Self-rated Health (SRH)** N (%) | Good | 414 (45.6%) | 70 (46.1%) | 484 (45.7%) |
|  | Moderate | 351 (38.7%) | 54 (35.5%) | 405 (38.2%) |
|  | Poor | 142 (15.7%) | 26 (17.1%) | 168 (15.9%) |
|  | Missing | 0 | 2 (1.3%) | 2 (0.2%) |
| **Medical Status** | | | | |
| **Body Mass Index (BMI)** (kg/m^2^) N (%) | ≤22 | 91 (10.0%) | 14 (9.2%) | 105 (9.9%) |
|  | 22-≤30 | 611 (67.4%) | 101 (66.4%) | 712 (67.2%) |
|  | >30 | 205 (22.6%) | 27 (17.8%) | 232 (21.9%) |
|  | Missing | 0 | 10 (6.6%) | 10 (0.9%) |
| **Charlson Comorbidity**  **Index (CCI)** N (%) | Median (IQR) | 7 (4,9) | 7 (5,9) | 7 (4,9) |
|  | 0 | 22 (2.4%) | 5 (3.3%) | 27 (2.5%) |
|  | 1-2 | 126 (13.9%) | 9 (5.9%) | 135 (12.7%) |
|  | 3-4 | 200 (22.1%) | 27 (17.8%) | 227 (21.4%) |
|  | ≥5 | 559 (61.6%) | 97 (63.8%) | 656 (61.9%) |
|  | Missing | 0 | 14 (9.2%) | 14 (1.3%) |
| **Polypharmacy** N (%) | Yes | 431 (47.5%) | 66 (43.3%) | 497 (46.9%) |
|  | Missing | 0 | 8 (5.3%) | 8 (0.8%) |
| **Frailty status** N (%) | Robust | 185 (20.4%) | 29 (19.1%) | 214 (20.2%) |
|  | Prefrail | 439 (48.4%) | 56 (36.8%) | 495 (46.7%) |
|  | Frail | 283 (31.2%) | 67 (44.1%) | 350 (33.1%) |

*Percentages of the social support categories are row percentages, whereas those of the individual variables are column percentages.

*CASMIN: Comparative Analysis of Social Mobility in Industrial Nations,

SRH: Self-rated Health,

BMI: Body Mass Index,

CCI: Charlson Comorbidity Index,

IQR: Interquartile Range

Table S3: Research question 2: Baseline characteristics stratified by inclusion status

| **Variable** | **Category** | **Included** | **Excluded** | **Total** N = 709 |
| --- | --- | --- | --- | --- |
|  |  | N = 588 (82.9%) | N = 121 (17.1%) |  |
| **Sociodemographic factors** | | | | |
| **Age**  Mean (SD) | | 82.7 (4.8) | 84.2 (5.6) | 82.9 (4.9) |
| **Gender**  N (%) | Female | 320 (54.4%) | 61 (50.4%) | 381 (53.7%) |
| **Social Support** N (%) | Strong | 187 (31.8%) | 42 (34.7%) | 229 (32.3%) |
|  | Moderate | 270 (45.9%) | 55 (45.5%) | 325 (45.8%) |
|  | Poor | 131 (22.3%) | 24 (19.8%) | 155 (21.9%) |
| **CASMIN** N (%) | High | 124 (21.1%) | 27 (22.3%) | 151 (21.3%) |
|  | Intermediate | 112 (19.0%) | 21 (17.4%) | 133 (18.8%) |
|  | Low | 352 (59.9%) | 70 (57.9%) | 422 (59.5%) |
|  | Missing | 0 | 3 (2.5%) | 3 (0.4%) |
| **Partner status** N (%) | Partnered | 341 (58.0%) | 60 (49.6%) | 401 (56.6%) |
|  | Missing | 0 | 1 (0.8%) | 1 (0.1%) |
| **Self-rated Health (SRH)** N (%) | Good | 330 (56.1%) | 68 (56.2%) | 398 (56.1%) |
|  | Moderate | 209 (35.5%) | 41 (33.9%) | 250 (35.3%) |
|  | Poor | 49 (8.3%) | 12 (9.9%) | 61 (8.6%) |
|  | Missing | 0 | 0 | 0 |
| **Medical Status** | | | | |
| **Body Mass Index (BMI)** (kg/m^2^) N (%) | ≤22 | 57 (9.7%) | 11 (9.1%) | 68 (9.6%) |
|  | 22-≤30 | 412 (70.1%) | 86 (71.1%) | 498 (70.2%) |
|  | >30 | 119 (20.2%) | 24 (19.8%) | 143 (20.2%) |
|  | Missing | 0 | 0 | 0 |
| **Charlson Comorbidity Index (CCI)** N (%) | Median (IQR) | 6 (4,8) | 7 (5,10) | 6 (4,8) |
|  | 0 | 19 (3.2%) | 5 (4.1%) | 24 (3.4%) |
|  | 1-2 | 107 (18.2%) | 7 (5.8%) | 114 (16.1%) |
|  | 3-4 | 153 (26.0%) | 27 (22.3%) | 180 (25.4%) |
|  | ≥5 | 309 (52.6%) | 71 (58.7%) | 380 (53.6%) |
|  | Missing | 0 | 11 (9.1%) | 11 (1.6%) |
| **Polypharmacy** N (%) | Yes | 235 (40.0%) | 49 (40.5%) | 284 (40.1%) |
|  | Missing | 0 | 6 (5.0%) | 6 (0.8%) |
| **Frailty status** N (%) | Robust | 180 (30.6%) | 34 (28.1%) | 214 (30.2%) |
|  | Prefrail | 408 (69.4%) | 87 (71.9%) | 495 (69.8%) |

*Percentages of the social support categories are row percentages, whereas those of the individual variables are column percentages.

*CASMIN: Comparative Analysis of Social Mobility in Industrial Nations,

SRH: Self-rated Health,

BMI: Body Mass Index,

CCI: Charlson Comorbidity Index,

IQR: Interquartile Range

Table S4: Multivariable multinomial regression model showing the association between social support and frailty transition categories in men.

|  | **Total**  **N = 398** | **Frailty Transition Categories** | | | | |
| --- | --- | --- | --- | --- | --- | --- |
|  |  | **Stable**  **Non-frail** | **Stable Frail** | **Improvement** | **Worsening** | **Death** |
| **Number of Events**  N (%) | | | | | | |
| **Social Support** | | | | | | |
| Strong | 129 | 56 (43.4%) | 13 (10.1%) | 16 (12.4%) | 22 (17.1%) | 22 (17.1%) |
| Moderate | 185 | 75 (40.5%) | 24 (13.0%) | 23 (12.4%) | 39 (21.1%) | 24 (13.0%) |
| Poor | 84 | 31 (36.9%) | 11 (13.1%) | 12 (14.3%) | 16 (19.0%) | 14 (16.7%) |
| **Crude Model**  RRR (95% CI) | | | | | | |
| **Social Support** | | | | | | |
| Moderate | | Reference | 1.38  (0.65 – 2.94) | 1.07  (0.52 – 2.22) | 1.32  (0.71 – 2.48) | 0.82  (0.42 – 1.60) |
| Poor | | Reference | 1.53  (0.61 – 3.82) | 1.36  (0.57 – 3.23) | 1.31  (0.60 – 2.86) | 1.15  (0.52 – 2.56) |
| **Adjusted Model**  RRR (95% CI) | | | | | | |
| **Social Support** | | | | | | |
| Moderate | | Reference | 1.05  (0.42 – 2.62) | 0.98  (0.46 – 2.09) | 1.15  (0.60 – 2.24) | 0.72  (3.34 – 1.54) |
| Poor | | Reference | 1.18  (0.39 – 3.57) | 1.22  (0.48 – 3.10) | 1.21  (0.53 – 2.78) | 1.07  (0.43 – 2.68) |

*The percentages in the descriptive part of the table are row percentages
*The model is adjusted for age, partner status, body mass index (BMI), Comparative Analysis of Social Mobility in Industrial Nations (CASMIN), self-rated health (SRH), polypharmacy, Charlson Comorbidity Index (CCI)

Table S5: Multivariable logistic regression model showing the association between social support and incident frailty in men.

|  | **Total**  **N =** **268** | **Incident Frailty** | | |
| --- | --- | --- | --- | --- |
|  |  | **Number of Events**  **N (%)** | **Crude**  **Model**  **OR (95% CI)** | **Adjusted Model**  **OR (95% CI)** |
| **Social Support** | | | | |
| Strong | 91 | 12 (13.2%) | Reference | Reference |
| Moderate | 125 | 21 (16.8%) | 1.33  (0.62 – 2.86) | 1.33  (0.56 – 3.19) |
| Poor | 52 | 12 (23.1%) | 1.98  (0.81 – 4.79) | 2.27  (0.82 – 6.29) |

*The model is adjusted for age, partner status, body mass index (BMI), Comparative Analysis of Social Mobility in Industrial Nations (CASMIN), self-rated health (SRH), polypharmacy, Charlson Comorbidity Index (CCI)

Table S6: Multivariable multinomial regression model showing the association between social support and frailty transition categories in women.

|  | **Total**  **N = 509** | **Frailty Transition Categories** | | | | |
| --- | --- | --- | --- | --- | --- | --- |
|  |  | **Stable**  **Non-frail** | **Stable Frail** | **Improvement** | **Worsening** | **Death** |
| **Number of Events**  N (%) | | | | | | |
| **Social Support** | | | | | | |
| Strong | 139 | 51 (36.7%) | 21 (15.1%) | 26 (18.7%) | 29 (20.9%) | 12 (8.6%) |
| Moderate | 234 | 79 (33.8%) | 43 (18.4%) | 48 (20.5%) | 44 (18.8%) | 20 (8.5%) |
| Poor | 136 | 48 (35.3%) | 31 (22.8%) | 14 (10.3%) | 25 (18.4%) | 18 (13.2%) |
| **Crude Model**  RRR (95% CI) | | | | | | |
| **Social Support** | | | | | | |
| Moderate | | Reference | 1.32  (0.70 – 2.48) | 1.19  (0.66 – 2.16) | 0.98  (0.55 – 1.76) | 1.08  (0.49 – 2.39) |
| Poor | | Reference | 1.57  (0.80 – 3.10) | 0.57  (0.27 – 1.22) | 0.92  (0.47 – 1.78) | 1.59  (0.70 – 3.66) |
| **Adjusted Model**  RRR (95% CI) | | | | | | |
| **Social Support** | | | | | | |
| Moderate | | Reference | 1.10 (0.53 – 2.30) | 1.17  (0.64 – 2.15) | 0.93  (0.51 – 1.69) | 1.01  (0.40 – 2.52) |
| Poor | | Reference | 1.46 (0.66 – 3.26) | 0.55  (0.25 – 1.19) | 0.82  (0.41 – 1.63) | 1.73  (0.65 – 4.63) |

*The percentages in the descriptive part of the table are row percentages
*The model is adjusted for age, partner status, body mass index (BMI), Comparative Analysis of Social Mobility in Industrial Nations (CASMIN), self-rated health (SRH), polypharmacy, Charlson Comorbidity Index (CCI)

Table S7: Multivariable logistic regression model showing the association between social support and incident frailty in women.

|  | **Total**  **N =** **320** | **Incident Frailty** | | |
| --- | --- | --- | --- | --- |
|  |  | **Number of Events**  **N (%)** | **Crude**  **Model**  **OR (95% CI)** | **Adjusted Model**  **OR (95% CI)** |
| **Social Support** | | | | |
| Strong | 96 | 13 (13.5%) | Reference | Reference |
| Moderate | 145 | 21 (14.5%) | 1.08  (0.51 – 2.28) | 1.01  (0.47 – 2.22) |
| Poor | 79 | 19 (24.1%) | 2.02  (0.93 – 4.41) | 2.04  (0.87 – 4.81) |

*The model is adjusted for age, partner status, body mass index (BMI), Comparative Analysis of Social Mobility in Industrial Nations (CASMIN), self-rated health (SRH), polypharmacy, Charlson Comorbidity Index (CCI)
